# Supplementary material for: Promising Markers of Inflammatory and Gut Dysbiosis in Patients with Post-COVID-19 Syndrome
Source: J Pers Med. 2023 Jun 8;13(6):971. doi: 10.3390/jpm13060971 (PMC10302977; doi:10.3390/jpm13060971)
Supplement: Supplementary file 1 [file jpm-13-00971-s001.zip › Supplementary S1. Operating Instructions.pdf]

Operating Instructions

1. PURPOSE

COLONOFLOR reagent kit is designed for the analysis of fecal samples by real-time PCR in order to quantify the microbiocenosis of the colon and detect dysbiotic disorders.

2. GENERAL INFORMATION

2.1. COLONOFLOR reagent kit helps to identify obligate representatives of the microflora of the large intestine (bifidobacteria, lactobacilli, escherichia coli), as well as conditionally pathogenic and pathogenic microorganisms (the list of detected microorganisms, depending on the kit pack type is stated in Table 4.

2.2. The scope of the kit is clinical laboratory diagnostics, scientific researches. Only for in vitro tests.

2.3. Target group of patients is men and women aged 14 years and older, belonging to the European population. Diagnostic significance of the test is to identify changes in the microbial composition of the intestine, indicating violation of the immune and metabolic balance.

2.4. Indication for prescription of the COLONOFLOR reagent kit is a need for assessment of the quantitative composition of the microbiota of the colon for diagnostic purposes or when conducting researches. Contraindication to the test is a period of less than 2 weeks after the antibiotic therapy. Conducting the study within this time frame is associated with the risk of obtaining unreliable results.

3. KIT FEATURE

3.1. REAGENT KIT FORMS

The reagent kit has three forms with different composition – Form 1 COLONOFLOR-8, Form 2 COLONOFLOR-16 (biocenosis) and Form 3 COLONOFLOR-16 (metabolism). The list of determined microorganisms for each form is given in Table 4.

Form 1 COLONOFLOR-8 is designed for 24 tests, including analysis of positive and negative control samples. The kit helps to identify 10 parameters, including 9 groups of microorganisms and the total bacterial number.

Form 2 COLONOFLOR-16 (biocenosis) is designed for 24 tests,
including an analysis of positive and negative control samples. The kit is able to identify 23 parameters, including 21 groups/species of microorganisms, the total bacterial number and the presence of pathogenicity genes that determine the enteroinvasive properties of E. coli.

Form 3 COLONOFLOR-16 (metabolism) is designed for 24 tests, including an analysis of positive and negative control samples. The kit is able to identify 19 parameters, including 18 groups of microorganisms and the total bacterial number.

3.2. COMPOSITION OF THE REAGENT KIT

The delivery package includes:

- reagent kit; instructions for use; certificate; file for quantitative processing of the results.

The reagent kit, depending on the form (Form 1 COLONOFLOR-8, Form 2 COLONOFLOR-16 (biocenosis), Form 3 COLONOFLOR-16 (metabolism)) is shown in Table 1-3.

Data on the specificity of the amplification mixtures for each form of the reagent kit rare stated in Table 4.

3.3. PRINCIPLE OF THE METHOD

Analysis of the composition of the microbial flora of fecal samples involves a two-stage study:

Stage 1 - DNA isolation;

Stage 2 - amplification of specific DNA sections by PCR with hybridization-fluorescence detection of amplification products in real time.

Table 1 **Form 1 COLONOFLOR-8**

Kit Component	Volume of the component, µl	Number of test tubes
Mixtures for amplification	20	24 strips of 8 test tubes each
Taq Polymerase Solution	1200	2 test tubes
Mineral oil	2000	3 test tubes
PCS Positive control sample	100	1 test tube
Negative control sample (NCS)	100	1 test tube

Table 2. **Form 2 COLONOFLOR-16 (biocenosis)**

Kit Component	Volume of the component, µl	Number of test tubes
Mixtures for amplification	20	48 strips of 8 test tubes each
Taq Polymerase Solution	1500	3 test tubes
Mineral oil	2000	5 test tubes
PCS-1 Positive control sample	200	1 test tube
PCS-2b Positive control sample	200	1 test tube
Negative control sample (NCS)	200	1 test tube

Table 3 **Form 3 COLONOFLOR-16 (metabolism)**

Kit Component	Volume of the component, µl	Number of test tubes
Mixtures for amplification	20	48 strips of 8 test tubes each
Taq Polymerase Solution	1500	3 test tubes
Mineral oil	2000	5 test tubes
PCS-1 Positive control sample	200	1 test tube
PCS-2m Positive control sample	200	1 test tube
Negative control sample (NCS)	200	1 test tube

Data analysis is performed using a file for quantitative processing of the results, which is a part of the kit. Calculation for each parameter will be performed using a formula describing the dependence of the threshold cycle Ct on the initial concentration of the DNA fragment. Conversion of the Ct threshold cycle values to the number of copies per ml will be carried out using Pfaffl method, adjusted for the efficiency of amplification. The obtained values (copies/ml) will be converted to CFU/ml, taking into account the copyicity of the gene used for amplification, and are the final result of the test. To get proper results, the version of the file attached to the set must be used.

COLONOFLOR reagent kit includes: a mixture for PCR-amplification specific to all bacteria (total bacterial mass); amplification mixtures specific to each detected species (or group) of bacteria (the list of detected microorganisms is state in Table 4).

The kit is intended for use on detection amplifiers for setting up a polymerase chain reaction in real time with the registration of a fluorescent signal through the FAM and HEX channels (DTLight, DT-prime (manufacturer DNA-Technology); CFX-96, MiniOpticon (manufactured by BioRad).

For quantitative processing of the test results obtained with the use of the COLONOFLOR reagent kit, you will need a file for processing that is part of the kit. After the end of the amplification, the indicators of the threshold (indicator) Ct cycle with the help of the file for processing will be converted into the number of each of the detected microorganisms, and the total bacterial number will be calculated in the same way. The obtained data reflect the state of the intestinal microbiocenosis, indicate the presence (absence) of dysbiotic shifts, as well as the presence of conditionally pathogenic flora. The results of the tests can be used in the complex diagnosis of diseases of the gastrointestinal tract.

Table 4. The list of detected microorganisms, composition of the strips and the channels for detection of the results of amplification.

Test tube no.	Name of the mix and channel for detection		Color of the mixture	Coating color
**COLONOFLOR-8**				
Test tube no.	Name of the mix and channel for detection		Color of the mixture	Coating color
	FAM	HEX		
1	Total bacterial mass		blue	colorless
2	Lactobacillus spp.		colorless	colorless
3	Bifidobacterium spp.		colorless	colorless
4	Escherichia coli		colorless	colorless
5	Bacteroides spp.		colorless	colorless
6	Faecalibacterium prausnitzii		colorless	colorless
7	Clostridium difficile	Klebsiella spp	colorless	colorless
8	Candida spp.	Staphylococcus aureus	colorless	colorless
**COLONOFLOR-16 (biocenosis)**				
**Strip 1**				
Test tube no.	Name of the mix and channel for detection		Color of the mixture	Coating color
	**FAM**	**HEX**		
1	Total bacterial mass		blue	colorless
2	Lactobacillus spp.		colorless	colorless
3	Bifidobacterium spp.		colorless	colorless
4	Escherichia coli		colorless	colorless
5	Bacteroides spp.		colorless	colorless
6	Faecalibacterium prausnitzii		colorless	colorless
7	Bacteroides thetaiotaomicron	Akkermansia muciniphila	colorless	colorless
8	Enterococcus spp		colorless	colorless
**Strip 2**				
Test tube no.	Name of the mix and channel for detection		Color of the mixture	Coating color
	**FAM**	**HEX**		
1	Escherichia coli enteropathogenic		blue	green
2	Klebsiella pneumonia	Klebsiella oxytoca	colorless	green
3	Candida spp.	Staphylococcus aureus	colorless	green
4	Clostridium difficile	Clostridium perfringens	colorless	green
5	Proteus vulgaris/mirabilis		colorless	green
6	Citrobacter spp.	Enterobacter spp.	colorless	green
7	Fusobacterium nucleatum	Parvimonas micra	colorless	green
8	Salmonella spp.	Shigella spp.	colorless	green
**COLONOFLOR-16 (metabolism)**				
**Strip 1**				
Test tube no.	Name of the mix and channel for detection		Color of the mixture	Coating color
	**FAM**	**HEX**		
1	Total bacterial mass		blue	colorless
2	Lactobacillus spp.		colorless	colorless
3	Bifidobacterium spp.		colorless	colorless
4	Escherichia coli		colorless	colorless
5	Bacteroides spp.		colorless	colorless
6	Faecalibacterium prausnitzii		colorless	colorless
7	Bacteroides thetaiotaomicron	Akkermansia muciniphila	colorless	colorless
8	Enterococcus spp		colorless	colorless
**Strip 2**				
Test tube no.	Name of the mix and channel for detection		Color of the mixture	Coating color
	**FAM**	**HEX**		
1	Blautia spp.		blue	pink

2	Acinetobacter spp.		colorless	pink
3	Streptococcus spp.		colorless	pink
4	Eubacterium rectale		colorless	pink
5	Roseburia inulinivorans		colorless	pink
6	Prevotella spp		colorless	pink
7	Methanobrevibacter smithii	Methanosphaera stadmanae	colorless	pink
8	Ruminococcus spp		colorless	pink

Figure 1. The scheme of strips for different forms of kits with the color of the amplification mixtures and the color of the coating substance.

Form 1 COLONOFLOR-8 Strip 1

Form 2 COLONOFLOR-16 (biocenosis) Strip 1

Strip 2

Form 3 COLONOFLOR-16 (metabolism) Strip 1

Strip 2

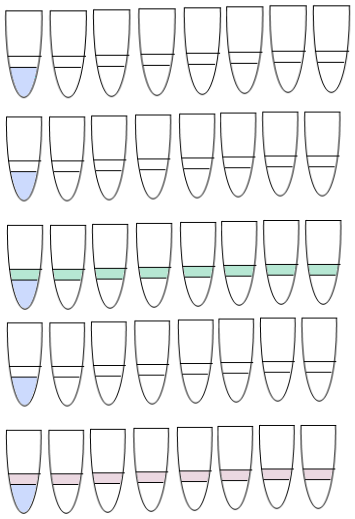

3.4. ANALYTICAL CHARACTERISTICS

3.4.1. Analytical sensitivity for individual microorganisms (groups of microorganisms) is:

	Mixture for amplification	Detection channel	Analytical sensitivity, copies/ml
1	Total bacterial mass	FAM	10^4
2	Lactobacillus spp.	FAM	10^5
3	Bifidobacterium spp.	FAM	10^5
4	Escherichia coli	FAM	10^5
5	Bacteroides spp.	FAM	10^5
6	Faecalibacterium prausnitzii	FAM	10^4
7	Clostridium difficile/Klebsiella spp	FAM/HEX	10^5/10^5
8	Candida spp/ Staphylococcus aureus	FAM/HEX	10^4/10^4
9	Bacteroides thetaiotaomicron/Akkermansia muciniphila	FAM/HEX	10^5/10^5
10	Enterococcus spp	FAM	10^5
11	Escherichia coli enteropathogenic	FAM	10^4
12	Klebsiella pneumoniae/Klebsiella oxytoca	FAM/HEX	10^5/10^5
13	Clostridium difficile/Clostridium perfringens	FAM/HEX	10^5/10^5
14	Proteus vulgaris/mirabilis	FAM	10^5
15	Citrobacter spp./Enterobacter spp.	FAM/HEX	10^5/10^5
16	Fusobacterium nucleatum/Parvimonas micra	FAM/HEX	10^5/10^5
17	Salmonella spp./Shigella spp.	FAM/HEX	10^4/10^4
18	Blautia spp.	FAM	10^5
19	Acinetobacter spp.	FAM	10^5
20	Streptococcus spp.	FAM	10^5
21	Eubacterium rectale	FAM	10^5
22	Roseburia inulinivorans	FAM	10^5

23	Prevotella spp	FAM	10^5
24	Methanobrevibacter smithii/ Methanosphaera stadmanae	FAM/HEX	10^5/10^5
25	Ruminococcus spp	FAM	10^5

Important: the kit corresponds to the specified analytical sensitivity values, provided that the quantitative values for PCR mixtures of all specificities calculated for positive control samples (PKO, PKO-1, PKO-2b and PKO-2m) using the file for processing correspond to those specified in the kit certificate.

3.4.2. Analytical specificity of the analysis made using the COLONOFLOR kit is confirmed:
- by absence of exponential growth of the fluorescent signal through the FAM and HEX channels in negative control samples;
- by sequencing the amplification products for each of the determined indicators;
- by absence of non-specific cross-reactions when testing DNA samples obtained from pure cultures of Lactobacillus spp., Bifidobacterium spp., Escherichia coli, Clostridium spp., Klebsiella pneumoniae, Klebsiella oxytoca, Candida albicans, Candida glabrata, Staphylococcus aureus, Enterococcus faecium, Enterococcus faecalis, Proteus mirabilis, Citrobacter freundii, Enterobacter spp., Salmonella spp., Streptococcus spp.

Thus, in the samples of biological material containing the DNA of the detected microorganism, the detecting amplifier during the amplification process must register an exponential increase in the level of fluorescence in the corresponding test tube (list of microorganisms and groups of microorganisms which can be detected by a kit is stated in Table 4). The samples of biological material that do not contain the DNA of the detected microorganism, showed no exponential increase in the level of fluorescence in the corresponding test tube during amplification.

4. SAFETY PRECAUTIONS

4.1. Potential risk of use of the kit - Class 2b

4.2. All components of the kit in the concentrations used are non-toxic.

4.3. Precautions - compliance with safety rules when working with microorganisms of groups III - IV of pathogenicity (danger) and pathogens of parasitic diseases.

4.4. Waste disposal will be carried out in accordance with the sanitary and epidemiological requirements for the treatment of medical waste, waste collection will be carried out using single-use bags intended for the disposal of medical waste of class B.

4.5. The laboratory process should be unidirectional. Separate rooms (zones) will be used for different stages of the analysis. The work should start in the isolation zone, continued in the amplification and detection zone. It is not allowed to return samples, equipment and reagents to the area where the previous stage of the process was carried out.

4.6. Only specially trained personnel are allowed to work with the kit.

4.7. It is allowed to use the kit only strictly for its intended purpose, according to these instructions and within the specified expiration date.

4.8. In the course of work, it is mandatory to use personal protective equipment: disposable gloves, lab coats. When working with the kit, avoid contact of the kit components with the skin, eyes and mucous membranes.

ATTENTION! When removing waste after amplification (test tubes containing PCR products), it is unacceptable to open the test tubes and spray the contents, since this can lead to contamination with PCR products of the laboratory area, equipment and reagents.

5. EQUIPMENT AND MATERIALS

Stage 1 - isolation of DNA from the fecal specimens.

Necessary equipment and materials:

- laminar flow box of the 2nd class of protection;
- centrifuge for test tubes with a capacity of 1.5 ml at 3,000-12,000 rpm

- microcentrifuge-vortex at 1,500-3,000 rpm;
- solid-state thermostat for test tubes with a capacity of 1.5 ml of the Termit type (DNA-Technology, Russia), which maintains a temperature of up to + 99°C;
- pipettes-dispensers of variable volume, which help you to select volumes of liquid 5-50; 20-200; 100-1,000 µl;
- disposable pipette tips with aerosol filter of 0.5-10; 20-200 µl; 100-1,000 µl
- container for dumping used tips, test tubes and other consumables;
- container with a cap for disinfectant solution;
- separate dressing gown, hats, shoes and gloves, rubber or plastic, disposable.
- **Stage 2** - PCR amplification and detection of amplification products

Required equipment:

- PCR box with UV lamp;
- "workplace" rack for stripped test tubes with a volume of 0.2 ml;
- microcentrifuge-vortex at 1,500-3,000 rpm;
- pipettes-dispensers of variable volume, which help you to select volumes of liquid 0.5-10; 5-50; 20-200 µl;
- disposable pipette tips with aerosol filter of 0.5-10; 20-200 µl;
- refrigerator with freezer for storing the initial reagents;
- separate lab coat, hats, shoes and gloves, rubber or plastic, disposable.
- container for dumping used tips, test tubes and other consumables;
- container with acap for disinfectant solution;
- programmable thermostat (amplifier) for PCR with fluorescence detection through two FAM and HEX channels in "real time" mode;
- software for analyzing the results.

6. TAKING, TRANSPORTING AND STORING THE TEST SAMPLES

6.1. Taking, transportation and storage of the test material should be carried out in strict accordance with the methodological recommendations for PCR-based diagnostics.

6.2. Test specimens are fecal samples. Fecal samples weighing 1-3 g (1-3 ml) are collected in a sterile plastic container. Smear testing is uninformative due to the low content of microorganisms.

6.3. The container with the material should be delivered to the laboratory and stored until the start of the analysis at 2-8 °C. The time from the collection of the material to the start of the analysis should not exceed 48 hours.

6.4. If it is necessary to store the material for a longer time, prepare a fecal suspension with glycerin, which is stored in the freezer - at a temperature not exceeding minus 18 °C for no more than 1 month, at a temperature of 70°C below zero for a long time. The procedure for preparing the fecal suspension is described in section 7.1.1. Repeated freezing and thawing of samples is not allowed.

7. RESTRICTIONS FOR THE USE OF THE ANALYZED MATERIAL

7.1. Before the test the samples of the biomaterial must pass the pre-treatment stage (see point 8.1. Preparation of the test material for DNA extraction).

7.2. For an adequate quantitative assessment of the composition of the intestinal microbiota, a strictly defined amount of biomaterial -0.1 g is taken for DNA isolation, that is, before the extraction procedure, the biomaterial must be weighed.

7.3. DNA extraction is carried out from the bacterial fraction of feces (p. 8.1.2.and 8.1.4), which provides an adequacy of the assessment of the intestinal microbiocenosis.

7.4. The means of control of adequate material collection and a qualitatively performed DNA extraction procedure are the indicators of the total bacterial mass not lower than 10^5 CFU/ml.

7.5. An excessive amount of material used for DNA isolation can result in inhibition of the PCR reaction and, accordingly, to distort the results of the analysis.

8. TEST CONDUCTION

8.1. PREPARATION OF THE TEST MATERIAL FOR DNA EXTRACTION

8.1.1. DNA extraction must be carried out from a liquid material, so feces of a watery consistency or fecal suspension should be used to isolate the DNA.

8.1.2. To prepare the fecal suspension, 0.8 ml of sterile isotonic sodium chloride solution is added to the appropriate number of microcentrifuge tubes (1.5 ml in volume). Next, 0.1 g of feces is added to each tube with a separate tip with an aerosol barrier (or disposable blades) and carefully resuspended on the vortex until a homogeneous suspension is formed.

8.1.3. If it is impossible to analyze the material during the day and/or there is a need for long-term storage, glycerin is added to the resulting fecal suspension in a final concentration of 10-15%. The mixture is thoroughly homogenized using a vertex and frozen after 30-40 minutes of exposure with glycerin.

8.1.4. Immediately before the DNA extraction procedure, the bacterial fraction of feces is prepared. To do this, test tubes with fecal suspension are centrifuged at 10,000 – 13,000 rpm for 30 seconds. The upper fraction (the supernatant) is used to isolate the DNA. The fecal suspension stored in the freezer must be kept at room temperature until it is completely defrosted before centrifugation.

8.1.5. When using watery feces, the suspension is not needed. 0.1 ml of feces is taken to sterile test tubes and centrifuged at 10,000 – 13,000 rpm for 30 seconds. The upper fraction (the supernatant) is used to isolate the DNA.

8.2. EXTRACTION OF DNA FROM THE FECAL SAMPLES

ATTENTION! The reagent kit for isolation of DNA from biological material is not included in the kit

8.2.1. For the purpose of DNA isolation, it is recommended to use reagent kits intended for use in clinical laboratory diagnostics for the analysis of fecal samples.

8.2.2. After the extraction procedure, the DNA samples should be stored at a temperature of 2 to 8 °C for no more than one week. Longer storage (up to 6 months) is possible at a temperature no higher than minus 18°C, and it is necessary to avoid repeated freezing/thawing of DNA samples.

8.3. PCR AMPLIFICATION

Total volume of the reaction mixture is 35 µl, including the volume of the DNA sample - 5 µl.

ATTENTION! Disposable tips with an aerosol barrier should be used to add reagents, DNA samples, and control samples to the test tubes!

8.3.1. Put the required number of strips* with a mixture for amplification (at the rate of n + 2, where n is the number of samples to be analyzed, two additional strips are intended for the analysis of PCS and NCS;

* Note: When using Form 1 COLONOFLOR-8, one strip must be prepared for each sample. When using Form 2 COLONOFLOR-16 (biocenosis) and Form 3 COLONOFLOR-16 (metabolism), two strips with different markings (strip-1, strip-2) must be prepared for each sample. The sequence of the test tubes in the strip is strict and shown in Table 3. For the purpose of correct orientation of the strips when placing them in the system, the mixture for amplification in the first tube of the strip is colored.

8.3.2. In each tube, without damaging the wax layer, add 10 µl of a thoroughly mixed solution of Taq polymerase;

8.3.3. Add a drop of mineral oil to each test tube;

8.3.4. Close the strip caps;

8.3.5. To prevent contamination, it is recommended to open the cap only of the stipe in which the sample will be inserted before adding the DNA, and close it before adding the next sample.

8.3.6. Add to each tube of a strip intended for a negative control sample, 5 µl of NCS;

8.3.7. Add in all test tubes of strips intended for the studied samples, 5 µl of the corresponding analyzed DNA sample;

8.3.8. Add in each tube of the strip intended for a positive control sample, 5 µl of PCS (for the form of the complete set Colonoflor-8);
8.3.9. For the form Colonoflor-16 (biocenosis) add to each test tube of strip 1, intended for a positive control sample, 5 µl of PCS-1; to each test tube of strip 2, intended for a positive control sample, add 5 µl of PCS-2b;
8.3.10. For the form Colonoflor-16 (metabolism), add 5 microliters of PCS-1 to each test tube of strip 1 intended for a positive control sample; add 5 microliters of PCS-2m to each tube of strip 2 intended for a positive control sample;
8.3.11. Install all the strips in the detection amplifier unit vertically, as shown in Figure 1, with the first (i.e., the colored tube) located on top. Fo the forms COLONOFLOR-16 (biocenosis) and COLONOFLOR-16 (metabolism) strips should be placed as follows: strip 1, strip 2, strip 1 of the subsequent sample, etc.;
8.3.12. Run the amplification program according to the instructions, for the device with the parameters specified in Table 5.
Figure 1. Position of the strips in the detecting amplifier unit.

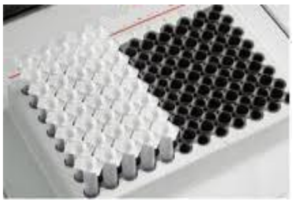

Table 5. Parameters of the amplification program

Amplification mode for CFX-96, MiniOpticon (BioRad) devices		
Temperature	Time	Number of cycles
94°C	15 min	1
94°C	5 sec	5
58°C	6 sec	
72°C	10 sec	
94°C	5 sec	40
58°C	6 sec *	
72°C	10 sec	
10°C - storage		
Amplification mode for Dtlight, DT-prime devices (DNA-Technology)		
Temperature	Time	Number of cycles
94°C	15 min	1
94°C	10 sec	45
58°C	10 sec *	
72°C	10 sec	
10°C - storage		

*** Fluorescence detection through FAM and HEX channels**

8.4. RECORDING OF AMPLIFICATION RESULTS

8.4.1. The fluorescent detection of amplification products is carried out in real time using a detecting amplifier.
8.4.2. After the end of the reaction, the detecting amplifier automatically generates a report on the study, including data on the value of the indicator cycle for each cell of the amplifier in which the device registered an increase in fluorescence.
8.4.3. The data contained in the test report is processed using a file for quantitative processing of the results of the analysis included in the set.

8.5. CONDUCTING THE TEST, ANALYZING AND INTERPRETING THE RESULTS - EXAMPLE DT96

8.5.1. Convenient start-up

8.5.1.1. Pre-create the COLONOFLOR test, taking into account the parameters of the amplification program specified in Table 4, according to the instructions for the device.

8.5.1.2. When running the test, enable the "Add test" option, select the created test COLONOFLOR, specify the number of patients (1), the number of duplicates, positive and negative controls-8 (for the option COLONOFLOR-8), 16 (for the option COLONOFLOR-16 (biocenosis and COLONOFLOR-16 (metabolism)):

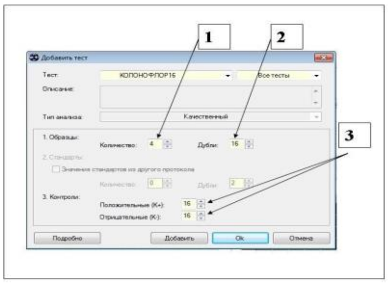

8.5.1.3. Mark the location of the strips on the heat block matrix in accordance with their installation by clicking the "Fill sequence" button:

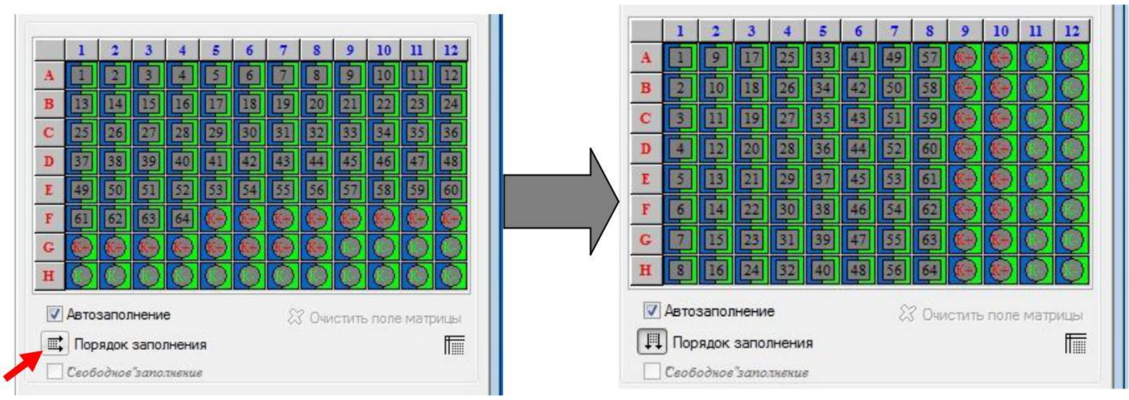

8.5.1.4. Then you can proceed to entering the names or identification numbers and start the amplification program

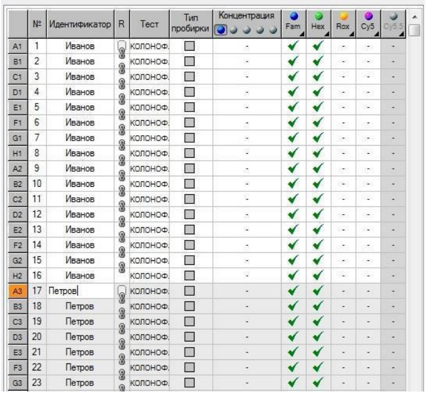

8.5.2. Analysis and interpretation of the results

8.5.2.1. At the end of the amplification program, select the Threshold (Ct) method and activate the "Report" menu:

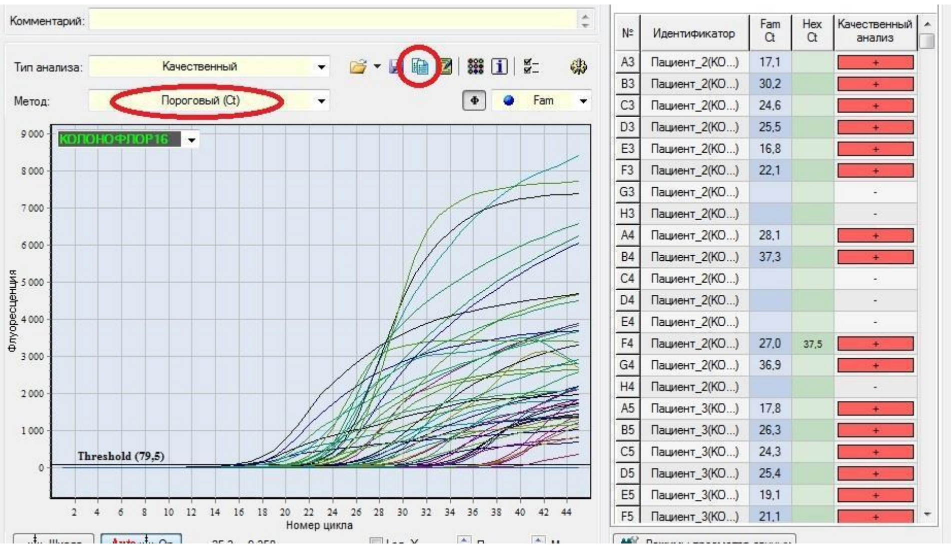

and save the report in the format *.rtf:

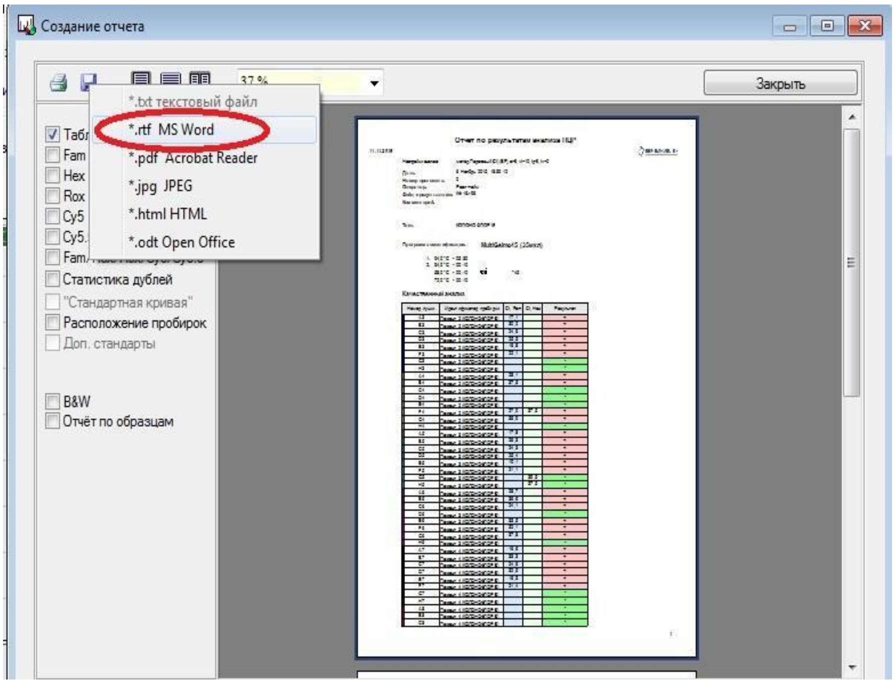

8.5.2.2. Run the file for quantitative processing of the results and load the received data into it.
8.5.2.3. Then in the top menu of the file you need to choose the type of the used instrument (detector of the amplifier) and the form of the used reagent kit – COLONOFLOR8, COLONOFLOR16 (biocenosis) or COLONOFLOR16 (metabolism) in the left bottom corner to enter patient data: the "Patient" is the name or identification number of the patient, filling the field "Age" required for the issuance of appropriate reference intervals in the conclusion.
8.5.2.4. Select the "Create Conclusion" mode in the menu, which you can then print or save:
8.5.2.5 For each sample conclusion is created automatically, and the conclusion reflects the following parameters:
qualitative and quantitative composition of obligate representatives of the microflora of the large intestine (bifidobacteria, lactobacilli, escherichia coli);
qualitative and quantitative composition of conditionally pathogenic flora (if found);
presence of dysbiotic shifts (the quantitative ratio of individual species (groups) of microorganisms);
The conclusion is formed on the basis of comparing the indicators obtained in the analysis of the clinical sample with the reference intervals for each group of microorganisms.
8.5.2.6. For each production series, it is necessary to compare the quantitative indicators for the PKO and OKO with the specified ones (specified in the passport for the set series), making an appropriate conclusion about the reliability of the test results.
8.5.2.7. When obtaining quantitative values for the PCS that exceed the tabular values by more than one order of magnitude relative to those specified in the certificate for the kit series, the results of the entire staged series are considered unreliable. All samples must be re-analyzed.
8.5.2.8. When the threshold cycle values are obtained below the maximum permissible values for the OKO specified in the passport for the kit series, the results of the entire staged series are considered unreliable. It is necessary to carry out a set of measures to eliminate contamination; after that, all samples of this series should be analyzed again.

8.5.3. Reference intervals[1]

8.5.3.1. Form 1 COLONOFLOR-8:

	Parameter	Reference interval (copies/ml)
1	Total bacterial mass	10^11-10^13
2	Lactobacillus spp.	10^7-10^8
3	Bifidobacterium spp.	10^9-10^10
4	Escherichia coli	10^6-10^8
5	Bacteroides spp.	10^9-10^12
6	Faecalibacterium prausnitzii	10^8-10^11
7	Ratio<br>Bacteroides spp./Faecalibacterium prausnitzii	0.01 - 100
8	Clostridium difficile	not found
9	Klebsiella spp	no more than 10^4
10	Candida spp.	no more than 10^4
11	Staphylococcus aureus	no more than 10^4

8.5.3.2. Form 2 **"COLONOFLOR-16 (biocenosis)":**

	Parameter	Reference interval (copies/ml)
1	Total bacterial mass	10^11-10^13
2	Lactobacillus spp.	10^7-10^8
3	Bifidobacterium spp.	10^9-10^10
4	Escherichia coli	10^6-10^8
5	Bacteroides spp.	10^9-10^12
6	Faecalibacterium prausnitzii	10^8-10^11

[1] Reference ranges for all forms of the kit (Form 1 COLONOFLOR-8, Form 2 COLONOFLOR-16 (biocenosis), Form 3 COLONOFLOR-16 (metabolism)) reflect the range of values for healthy donors (age group from 14 years) without gastrointestinal tract complaints.

7	Ratio<br>Bacteroides spp./Faecalibacterium prausnitzii ratio	0.01 - 100
8	Bacteroides thetaiotaomicron	Any quantity is allowed
9	Akkermansia muciniphila	any number up to 10^11 is allowed
10	Enterococcus spp	no more than 10^8
11	Escherichia coli enteropathogenic	no more than 10^4
12	Klebsiella pneumoniae	no more than 10^4
13	Klebsiella oxytoca	no more than 10^4
14	Candida spp.	no more than 10^4
15	Staphylococcus aureus	no more than 10^4
16	Clostridium difficile	not found
17	Clostridium perfringens	not found
18	Proteus vulgaris/mirabilis	no more than 10^4
19	Citrobacter spp.	no more than 10^4
20	Enterobacter spp.	no more than 10^4
21	Fusobacterium nucleatum	not found
22	Parvimonas micra	not found
23	Salmonella spp.	not found
24	Shigella spp.	not found

8.5.3.3. Form 3 COLONOFLOR-16 (metabolism)

	Parameter	Reference interval (copies/ml)
1	Total bacterial mass	10^11-10^13
2	Lactobacillus spp.	10^7-10^8
3	Bifidobacterium spp.	10^9-10^10
4	Escherichia coli	10^6-10^8
5	Bacteroides spp.	10^9-10^12
6	Faecalibacterium prausnitzii	10^8-10^11
7	Ratio<br>Bacteroides spp./Faecalibacterium prausnitzii ratio	0,01 - 100
8	Bacteroides thetaiotaomicron	Any quantity is allowed
9	Akkermansia muciniphila	any number up to 10^11 is allowed
10	Enterococcus spp	no more than 10^8
11	Blautia spp.	10^8-10^11
12	Acinetobacter spp.	not found
13	Eubacterium rectale	10^8-10^11
14	Streptococcus spp.	not found
15	Roseburia inulinivorans	any number up to 10^10 is allowed
16	Prevotella spp	any number up to 10^11 is allowed
17	Methanobrevibacter smithii	10^6-10^10
18	Methanosphaera stadmanae	no more than 10^6
19	Ruminococcus spp	any number up to 10^11 is allowed

9. CONDITIONS OF TRANSPORTATION, STORAGE AND OPERATION OF THE KIT

9.1. Shelf life of the reagent kit is 6 months from the date of manufacture.

9.2. Storage conditions for individual components of the kit reagents COLONOFLOR are stated on the packaging. Strips with mixtures for amplification should be stored in a place protected from light at a temperature of + 2 to + 8 °C. The Taq polymerase solution must be stored at a temperature of + 2 to + 8 °C.

9.3. To obtain reliable results, you must strictly follow the kit operational instructions.
