# Supplementary material for: Promising Markers of Inflammatory and Gut Dysbiosis in Patients with Post-COVID-19 Syndrome
Source: J Pers Med. 2023 Jun 8;13(6):971. doi: 10.3390/jpm13060971 (PMC10302977; doi:10.3390/jpm13060971)
Supplement: Supplementary file 1 [file jpm-13-00971-s001.zip › Supplementary S2. Questionnaire for COVID-19 Survivors on Admission.pdf]

A Questionnaire for COVID-19 Survivors on Admission

Second name ☐

First name ☐

Date of Birth ☐

Phone number ☐

e-mail ___________________________________

Time of onset of symptoms of coronavirus infection (date)

☐

Date of discharge ____

COVID-19 test status now

☐ Negative PCR, date of determination «_____» _______________

Date you were diagnosed with COVID-19 ☐

What symptoms of coronavirus infection were noted during the acute phase of the disease?

☐ Temperature (specify what was) _________________________________

☐ Shortness of breath

☐ Cough

☐ Loss of smell (anosmia) or loss of taste (ageusia)

☐ General weakness

☐ Myalgia

☐ Gastrointestinal intestinal symptoms

Have you been hospitalized?

○ Yes

○ No

Have you been diagnosed with pneumonia?

○ Yes. The result of the CT scan____________ before hospitalization. CT_________ at discharge

○ No

Was there breathing support for respiratory failure?

○ Yes. What and how many days?

____

◉ No

What is bothering you now?

Headache, dizziness?

Yes

No

Loss of taste and smell?

Yes

No

Episodes of excessive sweating, high blood pressure, palpitations, cold hands and feet?

Yes

No

Do you have a cough?

Yes

No

Do you feel short of breath, feeling short of breath at rest?

Yes

No

- during increased physical activity

Yes

No

Are you experiencing problems with gastrointestinal disorders?

Yes

No

Do you suffer from muscle pain?

Yes

No

Do you suffer from excessive daytime sleepiness and fatigue?

Yes

No

Are you concerned about sleep disorders?

Yes

No

Do you have memory and attention disorders?

Yes

No

Do you have weakness in your arms or legs that interferes with your daily activities?

Yes

No

Do you have numbness of the face, body, upper or lower extremities?

- ○ Yes
- ○ No

If your symptoms are not listed, please describe them in more detail.____
____
____
____
____
______

Do you have chronic diseases?____
____
____
____
____
____

Patient's signature ________________________/ _______________________________

Specialist signature ____________________/________________________________
