# Supplementary material for: Promising Markers of Inflammatory and Gut Dysbiosis in Patients with Post-COVID-19 Syndrome
Source: J Pers Med. 2023 Jun 8;13(6):971. doi: 10.3390/jpm13060971 (PMC10302977; doi:10.3390/jpm13060971)
Supplement: Supplementary file 1 [file jpm-13-00971-s001.zip › Supplementary Tables S1-S6 (REVISED).pdf]

Journal of
Personalized
Medicine

MDPI

Supplementary Tables for "Promising Markers of Inflammatory and Gut Dysbiosis in Patients with Post-COVID-19 Syndrome"

Table S1. Retrospective CT scan data during acute COVID-19 (lung injury, %) and CT scan data in patients (n = 20) after 14 days of rehabilitation.

Patient, sex	Retrospective CT Scan Data, Lung Injury, %	After 14 days of rehabilitation				
		Lung Injury, %	Lung Capacity, cm3	Lung Injury, cm^3	Left Lung Injury, cm^3	Right Lung Injury, cm^3
Patient #1, female	20	1	4512	34	20	14
Patient #2, female	25	4	3607	126	59	67
Patient #3, male	40	1	6153	59	25	34
Patient #4, female	25	6	3229	179	62	117
Patient #5, male	55	25	4497	1119	578	540
Patient #6, female	10	1	4668	40	16	24
Patient #7, female	80	30	2700	783	418	366
Patient #8, male	40	2	6246	101	53	54
Patient #9, female	15	13	3019	367	246	121
Patient #10, female	25	11	3759	400	226	174
Patient #11, female	15	12	3345	386	175	211
Patient #12, male	44	2	6130	138	63	76
Patient #13, female	54	14	3531	494	209	285
Patient #14, female	25	1	3790	27	18	9
Patient #15, female	70	26	3220	800	436	364
Patient #16, female	50	17	3236	521	286	235
Patient #17, male	25	8	4702	390	265	125
Patient #18, male	13	3	8016	117	65	52
Patient #19, male	5	3	4741	121	48	73
Patient #20, male	53	9	5148	461	196	265
Median, 25-75%	25 (18–52)	7 (2–14)	*	*	*	*

* Reference values, the median and IQR are different for male/female

Table S2. The results of the complete blood count in patients with post-COVID-19 syndrome (*n* = 39) on the day of the admission for the rehabilitation. Reference values are combined for male/female, but *n* (c > RV) / *n* (c < RV) indicates the number of samples with a higher / lower level than the corresponding reference value taking into account the difference in the reference values for male/female.

Parameter	Reference Values	Patients on the admission (*n* = 39)
White Blood Cell Count (WBC), ×10⁹/L	3.8–11.8	5.6 (5.3–6.8), 3.0–10.9<br>*n* (c < RV) = 1
Red Blood Cell Count (RBC), ×10¹²/L	3.63–5.63	4.59 (4.26-5.02), 3.71–5.56<br>*n* (c > RV) = 6
Hemoglobin (Hb), g/L	109–163	134 (125-146), 95–169<br>*n* (c > RV) = 7<br>*n* (c < RV) = 1
Haematocrit (Hct), %	31.2–47.1	40.5 (37.7-44.1), 31.1–49.6<br>*n* (c > RV) = 9<br>*n* (c < RV) = 1
Mean Cell Volume (MCV), fL	75.5–95.3	88.6 (86.2–91.6), 62.2–96.9<br>*n* (c > RV) = 3<br>*n* (c < RV) = 1
Mean Cell Hemoglobin (MCH), pg/cell	24.7–33.4	29.5 (28.4–30.3), 19.0–33.7<br>*n* (c > RV) = 1<br>*n* (c < RV) = 1
Mean Cell Hemoglobin Concentration (MCHC), g/L	323–356	332 (326–335), 305–348<br>*n* (c < RV) = 4
Red Blood Cell Distribution Width (RDW), %	12.3–17.7	14.0 (13.6–15.0), 10.2–20.7<br>*n* (c > RV) = 2<br>*n* (c < RV) = 2
Platelet Count (Plt), ×10⁹/L	179–408	240 (200–298), 72–436<br>*n* (c > RV) = 1<br>*n* (c < RV) = 3
Mean Platelet Volume (MPV), fL	7.9–10.8	9.2 (8.4–9.9), 7.5 -10.8<br>*n* (c < RV) = 1
Neutrophils, %	42.7–76.8	55.3 (51.3–61.3), 37.4–71.3<br>*n* (c < RV) = 2
Lymphocytes, %	16.0–45.9	32.4 (27.7–38.2), 18.6–48.8<br>*n* (c > RV) = 3
Monocytes, %	4.3-10.9	8.3 (7.2–9.7), 5.9–12.7<br>*n* (c > RV) = 3
Eosinophils, %	0.5–7.0	2.5 (1.6–3.6), 0.9–8.8<br>*n* (c > RV) = 1
Basophils, %	0.2–1.3	0.8 (0.6–1.0), 0.1–1.7<br>*n* (c > RV) = 2
Absolute Neutrophils, ×10⁹/L	1.9-8.2	3.3 (2.9–3.9), 1.7–6.2<br>*n* (c < RV) = 2
Absolute Lymphocytes, ×10⁹/L	1.1–3.1	1.9 (1.6–2.4), 0.8–3.5<br>*n* (c > RV) = 2<br>*n* (c < RV) = 2
Absolute Monocytes, ×10⁹/L	0.2–0.9	0.5 (0.4–0.6), 0.3–0.8
Absolute Eosinophils, ×10⁹/L	<0.5	0.2 (0.1–0.2), 0.1–0.6<br>*n* (c > RV) = 1
Absolute Basophils, ×10⁹/L	<0.10	0.01 (<0.10–0.10), <0.10–0.10

Erythrocyte Sedimentation Rate (ESR), mm/hr	<20.0	14.0 (8.0–23.5), 3.0–37.0<br>*n* (c > RV) = 10

Table S3. The results of the coagulation test in patients with post-COVID-19 syndrome (*n* = 39) on the day of the admission for the rehabilitation. *n* (c > RV) / *n* (c < RV) indicates the number of samples with a higher / lower level than the corresponding reference value.

Parameter	Reference Values	Patients on the Admission (*n* = 39)
Prothrombin Time (PT), sec	9.4–12.5	11.1 (10.5–11.7), 9.4–16.4<br>*n* (c > RV) = 6
Prothrombin by Quik,%	80–140	109 (85–132), 43–167<br>*n* (c > RV) = 3<br>*n* (c < RV) = 4
International Normalised Ratio (INR)	0.90–1.20	1.03 (0.95–1.10), 0.86–1.55<br>*n* (c > RV) = 5<br>*n* (c < RV) = 1
Fibrinogen Activity, g/L	2.38–4.98	3.29 (2.58–3.63), 1.67–6.08<br>*n* (c > RV) = 1<br>(c < RV) = 5
Activated Partial Thromboplastin Time (PTT), sec	25.0–36.5	29.6 (27.8–33.1), 23.3–57.0<br>*n* (c > RV) = 6<br>*n* (c < RV) = 2
Thrombin Time (TT), sec	11.0–17.8	17.2 (14.0–17.7), 12.1–19.6<br>*n* (c > RV) = 7
D-dimer, μ/mL	<0.49	0.22 (0.14–0.39), 0.07–1.89<br>*n* (c > RV) = 5

Table S4. The results of the blood chemistry test, the concentrations of interleukin-6 and neuronspecific enolase in patients with post-COVID-19 syndrome (*n* = 39) on the day of the admission for the rehabilitation. *n* (c > RV) / *n* (c < RV) indicates the number of samples with a higher / lower level than the corresponding reference value.

Parameter	Reference Values	Patients on the Admission (*n* = 39)
Bilirubin, μmol/L	5.0–21.0	11.3 (8.5–17.0), 3.5–38.1<br>*n* (c > RV) = 5<br>*n* (c < RV) = 1
Total Protein, g/L	66.0–83.0	69.8 (66.7–72.3), 62.1–81.2<br>*n* (c < RV) = 1
Creatinine, μmol/L	58.0–110.0	86.5 (79.8–95.4), 69.3–166.0<br>*n* (c > RV) = 3
Glucose, mmol/L	4.1–5.9	5.3 (5.0–5.9), 4.1–10.5<br>*n* (c > RV) = 7
Cholesterol, mmol/L	<5.2	5.2 (4.2–6.1), 2.4–8.4<br>*n* (c > RV) = 19
Lactate Dehydrogenase (LDH), U/L	<247.0	192.9 (156.6–215.9), 131.4–306.3<br>*n* (c > RV) = 4
Alanine Transaminase (ALT), U/L	<50.0	18.7 (14.9–25.8), 9.2–44.5<br>*n* (c > RV) = 2
Aspartate Transaminase (AST), U/L	<50.0	21.1 (18.7–24.1), 15.5–91.7<br>*n* (c > RV) = 3

C-Reactive Protein (CRP), mg/L	<5.0	0.6 (0.1–0.9), 0.1–30.1<br>*n* (c > RV) = 8
Uric acid, µmol/L	154.7–428.0	313.9 (254.0–389.3), 196.5–528.0<br>*n* (c > RV) = 10
Interleukin-6 (IL-6), pg/mL	<7.0	10.9 (7.9–15.0), 1.5–61.9<br>*n* (c > RV) = 33
Neuronspecific Enolase (NSE), ng/mL	<16.0	8.0 (3.6–11.6), 0.1–23.0<br>*n* (c > RV) = 3

Table S5. The concentrations of metabolites in the serum samples of the healthy volunteers (*n* = 48) and patients with post-COVID-19 syndrome (*n* = 39) on the day of the admission for the rehabilitation, and the results of the Mann-Whitney U-test. *n* (c > 0.5) indicates the number of samples with a level above the limit of quantitation.

Acid, µmol/L	Healthy Volunteers (*n* = 48)	Patients on the admission (*n* = 39)	*p*-Value
Benzoic	<0.5 (<0.5–<0.5), <0.5–0.6<br>*n* (c > 0.5) = 2	<0.5 (<0.5-<0.5), <0.5–0.8<br>*n* (c > 0.5) = 7	-
Phenylpropionic	<0.5 (<0.5–0.5), <0.5–3.0<br>*n* (c > 0.5) = 15	<0.5 (<0.5–0.6), <0.5–5.1<br>*n* (c > 0.5) = 15	-
Phenyllactic	<0.5 (<0.5–<0.5), <0.5–0.7<br>*n* (c > 0.5) = 2	not detected	-
4-Hydroxybenzoic	not detected	6.6 (4.1–8.8), 1.0–13.0<br>*n* (c > 0.5) = 39	**<0.001**
4-Hydroxyphenylacetic	<0.5 (<0.5–<0.5), <0.5–1.2<br>*n* (c > 0.5) = 5	<0.5 (<0.5–0.5), <0.5–1.6<br>*n* (c > 0.5) = 12	-
4-Hydroxyphenyllactic	1.2 (0.9–1.5), 0.7–2.5<br>*n* (c > 0.5) = 48	1.1 (0.9–1.3), 0.6–2.7<br>*n* (c > 0.5) = 39	0.166
Succinic	4.8 (4.4–6.0), 3.3–12.4<br>*n* (c > 0.5) = 48	11.8 (9.0–15.0), 7.1–25.0<br>*n* (c > 0.5) = 39	**<0.001**
Fumaric	1.3 (1.1–1.5), 0.8–2.3<br>*n* (c > 0.5) = 48	2.2 (1.7–2.9), 1.0–5.5<br>*n* (c > 0.5) = 39	**<0.001**

Table S6. Taxonomic abundance of the gut microbiota in patients with post-COVID-19 syndrome (*n* = 39) on the day of the admission for the rehabilitation. *n* (c > $10^4/10^5$) indicates the number of samples with a level above the limit of quantitation. Reference values (RV) were obtained for the healthy volunteers without gastrointestinal complaints (age over 14 years). *n* (c > RV) / *n* (c < RV) indicates the number of samples with a higher / lower level than the corresponding reference value.

Parameter	Reference Values	Patients on the Admission (*n* = 39)
Total bacterial mass	10^{11}–10^{13}	$2×10^{13}$ ($9×10^{12}$–$7×10^{13}$), $6×10^{11}$–$4×10^{15}$<br>*n* (c > 10^4) = 39<br>*n* (c > RV) = 24
Lactobacillus spp.	10^7–10^8	$4×10^7$ ($3×10^6$–$3×10^8$), $1×10^5$–$4×10^{10}$<br>*n* (c > 10^5) = 39<br>*n* (c > RV) = 13<br>*n* (c < RV) = 14
Bifidobacterium spp.	10^9–10^{10}	$3×10^{10}$ ($1×10^{10}$–$1×10^{11}$), $2×10^8$–$3×10^{12}$<br>*n* (c > 10^5) = 39<br>*n* (c > RV) =27<br>*n* (c < RV) = 2
Escherichia coli	10^6–10^8	$2×10^8$ ($2×10^7$–$2×10^9$), $2×10^6$–$5×10^{11}$<br>*n* (c > 10^5) = 39

		n (c > RV) = 22
Bacteroides spp.	10^9–10^{12}	2×10^{13} (9×10^{12}–7×10^{13}), 6×10^{11}–4×10^{15}<br>*n* (c > 10^4) = 39<br>*n* (c > RV) = 37
Faecalibacterium prausnitzii	10^8–10^{11}	9×10^{10} (4×10^{10}–5×10^{11}), 1×10^7–5×10^{13}<br>*n* (c > 10^4) = 39<br>*n* (c > RV) = 19<br>*n* (c < RV) = 1
Bacteroides thetaiotaomicron	>10^4	5×10^9(1×10^8–2×10^{10}), 4×10^6–4×10^{11}<br>*n* (c > 10^5) = 28
Akkermansia muciniphila	<10^{11}	2×10^7 (2×10^7–7×10^{10}), 1×10^5–3×10^{12}<br>*n* (c >10^5) = 7
Enterococcus spp.	<10^8	<10^5 (<10^5–<10^5), <10^5–4×10^{12}<br>*n* (c >10^5) = 1<br>*n* (c > RV) = 1
Escherichia coli enteropathogenic	<10^4	<3×10^6 (5×10^4–5×10^6), 5×10^4–5×10^6<br>*n* (c >10^4) = 2<br>*n* (c > RV) = 2
Candida spp.	<10^4	1×10^6 (7×10^5–2×10^6), 1×10^5–3×10^7<br>*n* (c >10^4) = 5<br>*n* (c > RV) = 5
Klebsiella oxytoca	<10^4	not detected
Staphylococcus aureus	<10^4	6×10^6 (3×10^5–2×10^7), 2×10^5–5×10^7<br>*n* (c >10^4) = 11<br>*n* (c > RV) = 11
Clostridium difficile	not detected	not detected
Clostridium perfringens	not detected	3×10^6 (2×10^6–1×10^7), 2×10^6–1×10^7<br>*n* (c >10^5) = 3<br>*n* (c > RV) = 3
Proteus vulgaris/mirabilis	<10^4	5×10^6 (4×10^5–1×10^7), <10^5–2×10^9<br>*n* (c >10^5) = 5<br>*n* (c > RV) = 5
Enterobacter spp.	<10^4	2×10^7 (3×10^6–2×10^8), 1×10^5–7×10^{10}<br>*n* (c >10^5) = 26<br>*n* (c > RV) = 26
Citrobacter spp.	<10^4	5×10^7 (3×10^6–3×10^{13}), 2×10^5–5×10^{13}<br>*n* (c >10^5) = 4<br>*n* (c > RV) = 4
Fusobacterium nucleatum	not detected	7×10^5 (2×10^5–5×10^6), 1×10^5–1×10^7<br>*n* (c >10^5) = 7<br>*n* (c > RV) = 7
Parvimonas micra	not detected	2×10^5 (1×10^5–1×10^6), 1×10^5–2×10^6<br>*n* (c >10^5) = 4<br>*n* (c > RV) = 4
Salmonella spp.	not detected	not detected
Shigella spp.	not detected	not detected
Bacteroides fragilis /*Faecalibacterium prausnitzii* Ratio	0.01–100	133 (75–1000) 1.14–40000<br>*n* (c > RV) = 14
